# Effect of 2 Weeks Naringin Application on Neurological Function and Neurogenesis After Brain Ischemia–Reperfusion in Ovariectomized Rats

**DOI:** 10.1007/s12035-025-05050-w

**Published:** 2025-05-15

**Authors:** Aysenur Keskin, Gozde Acar, Tugce Aladag, Ummugulsum Onal, Saltuk Bugra Baltaci, Rasim Mogulkoc, Abdulkerim Kasim Baltaci

**Affiliations:** 1 Faculty of Medicine, Department of Physiology, Selcuk University, Konya, Turkey; 2 Faculty of Medicine, Department of Histology, Selcuk University, Konya, Turkey; 3 Faculty of Medicine, Department of Physiology, Istanbul Medipol University, Istanbul, Turkey

**Keywords:** Brain ıschemia-reperfusion, Neurological score, Calbindin, Tubulin, Neu-N, Naringin

## Abstract

Cerebral ischemia–reperfusion (I/R) is a condition that occurs when blood flow is restored after a temporary interruption and may lead to deterioration in brain functions depending on the time passed. One of the changes in functions is neurological score values. This study aimed to determine the effect of brain ischemia reperfusion and 2-week naringin supplementation on changes in neurological score and neurogenesis in ovariectomized female rats. Experimental groups of 36 Wistar-albino-type female rats were created as follows: control group: no anesthesia or surgical procedure was applied. Ovariectomy-sham brain I/R group: After the ovariectomy was performed under general anesthesia, the carotid artery regions were opened and closed, and sham ischemia–reperfusion was performed, followed by a vehicle application for 2 weeks (2 weeks, 1 ml 0.25% carboxymethylcellulose). Ovariectomy-I/R group: After ovariectomy, carotid arteries were isolated under general anesthesia, ligated for 30 min, and reperfused for 2 weeks after ischemia was performed. Ovariectomy-I/R sham treatment group: After ovariectomy, the carotid arteries were isolated under general anesthesia, then ligated and ischemia was performed for 30 min, and then reperfusion and vehicle application were performed for 2 weeks. Ovariectomy-I/R naringin treatment group: After ovariectomy, carotid arteries were isolated under general anesthesia, ligated for 30 min, and ischemia was performed, followed by naringin application with reperfusion for 2 weeks. Neurological scoring values performed on the 1st, 7th, and 14th days after the surgical procedure significantly increased with ischemia–reperfusion. Also, hippocampus and frontal cortex calbindin, alpha/beta-tubulin, and Neu-N levels were reduced considerably by ischemia–reperfusion. However, it was observed that a 2-week naringin application significantly suppressed the increase in neurological scores. The suppression in neurological score values became more evident in the 2nd week. Our results show that the impairment of motor functions and neurogenesis in the frontal cortex and hippocampus in brain ischemia–reperfusion after ovariectomy in female rats was significantly improved by 2 weeks of naringin supplementation.

## Introduction

Restricting blood flow to an organ or tissue is defined as ischemia, and subsequent restoration of blood flow and oxygen is described as perfusion [1]. With the decrease or stop of arterial blood flow in ischemia, metabolic imbalance occurs and tissue hypoxia develops [2]. Although restoration of reperfusion is the most effective way to reduce ischemic damage, it causes increased tissue damage and a pathological condition by creating an inflammatory response [3].

Ischemia–reperfusion (I/R) is associated with a vascular phenotype that includes increased blood flow, endothelial cell inflammation, an imbalance between vasodilator and vasoconstrictor factors, and activation of the coagulation and complement systems [2]. As a result of I/R, oxidative damage, endothelial barrier dysfunction, mitochondrial dysfunction, cell death, excessive calcium increase, inflammation, and autoimmune responses are observed [4]. Reactive oxygen species (ROS) production begins in the early period of ischemia, and with reperfusion, excessive oxidative stress occurs, causing cell death [5]. I/R occurs in a sterile environment, but innate and adaptive immune responses are also activated and contribute to damage [2]. I/R in the brain, heart, kidney, lung, and liver causes fatal consequences [1].

In I/R, it causes cell death through apoptosis, autophagy, and necrosis [6]. Autophagy is cell death caused by cytoplasmic vacuolization, organelle loss, and accumulation of membrane-folded vacuoles [2]. In necrosis, while it is characterized by cell and organelle swelling, rupture of surface membranes, and cell contents coming out of the cell, apoptosis is characterized by cell and nuclear shrinkage induced by caspase signaling and maintenance of plasma membrane integrity [6].

Neurogenesis is a developmental process that ensures the balance between neural stem cells (NSCs) by passing through successive stages such as self-renewal, proliferation, and differentiation of NSCs into neurons [7]. NSCs proliferate to increase their population, migrate to certain parts of the brain, differentiate there, and the newly formed cells join the circuit and mature [8].

In neurological research, the expression level of neuronal nuclei (NeuN) is used to evaluate neuron death [9]. In a study, it was confirmed that hesperidin flavonoid treatment prevented the decrease in neural cell survival, along with the improvement in NeuN-positive cell loss in rats with hippocampal neurogenesis-related memory impairment [10].

After ovariectomy, estrogen and progesterone levels in females decrease, causing depressive-like behaviors, changes in glucose metabolism in brain regions related to cognition and emotion, deterioration of somatic features, amyloid accumulation, and morphological changes in the cholinergic system [11].

Alpha and beta tubulins are two highly conserved proteins within the tubulin superfamily [12]. They play vital roles in numerous cellular processes, including intracellular traffic, migration, and mitosis, and alpha and beta tubulin heterodimers form microtubules. Alpha I tubulin (TUBA1 A) is generally absent in the proliferative ventricular zone during development [13]. In adulthood, it is expressed in various neural structures and in the subgranular zone (SGZ) and subventricular zone (SVZ) [14]. TUBA1 A is expressed in type-4 and type-5 cells, but not in type-1, type-2, and type-3 cells [13]. TUBB3 is expressed only in neurons and is not found in glial and other supporting cells. It makes microtubules more dynamic than other beta-isotypes [15]. TUBB3 expression is associated with the earliest stages of neuronal differentiation; it is at a high level in the cortex in the first postnatal period and decreases over time with increasing postnatal development [16].

Calbindin is a cytosolic calcium-binding protein that protects neurons from harmful factors and regulates the intracellular calcium concentration in neurons through its buffering capacity [17]. Calbindin, which binds to cellular structures in neurons, is a protein that plays a role in the regulation of calcium pools, which is important for synaptic flexibility [18]. It prevents neuron death by inhibiting different pro-apoptotic pathways [19].

Flavonoids prevent DNA damage by suppressing oxidative stress [20]. Thus, they provide effective treatment in the treatment of cerebrovascular diseases and ischemia-induced brain damage [21]. Flavonoids exert their anti-inflammatory effects by altering intracellular signaling pathways such as nuclear factor kappa B, JAK-STATs, and mitogen-activated protein kinases (MAPKs) [22]. Flavonoids are effective in gluco-regulation by increasing insulin secretion, reducing apoptosis, promoting β-cell profiling, reducing insulin resistance in muscles and other cells, and reducing oxidative stress [23]. Flavonoids affect cognition and delay cognitive aging [24].

Studies show that flavonoids have neuroprotective effects against ischemic damage. A study showed that flavonoid protects from I/R damage by demonstrating an anti-oxidative effect, improving neurobehavioral, neurochemical, and histological aspects [25]. In another study, flavonoids showed neuroprotective effects in rats with cerebral I/R injury by inhibiting protein kinases, increasing blood flow to the tissue, and reducing the delayed death of pyramidal cells [26].

Naringin (naringenin 7-O-β-rhamnoglycoside) is a flavonoid (Tripoli et al., 2007), which has been shown to induce the migration of NSCs and accelerate neuronal maturation by promoting neuronal differentiation in a depression model [27].

The current study aimed to determine the effect of 2-week naringin supplementation on neurological function and effective factors in neurogenesis after focal brain ischemia–reperfusion in ovariectomized rats**.**

## Materials and Methods

This study was conducted on 36 Wistar-albino type female rats, weighing between 230 and 330 g (290 g), 10–12 weeks old, obtained from the same center, with the approval number 2023–03 received from the Ethics Committee of Selçuk University Experimental Medicine Application and Research Center on 26/05/2023 that was carried out. One week before the study, they were fed a standard diet and as much water as they wanted. Rats were kept in a 12-h-light/12-h-dark cycle at an average temperature of 22 ± 2 °C.

Brain I/R surgery was performed 3 days after ovariectomy was performed on the rats. Following the operation, naringin treatment and vehicle supplementation were applied for 14 days. Neurological scoring was performed on the 1 st, 7 th, and 14 th days of treatment. After the 14 th day of treatment, the rats were sacrificed by cervical dislocation. Hippocampus and frontal tissues taken from rats were stored at − 80 °C until the availability of appropriate analysis kits and chemicals. Molecular analyses of the study were performed in the Selçuk University Faculty of Medicine Molecular Physiology Laboratory, and histological analyses were performed in the Selçuk University Faculty of Medicine Histology Laboratory.

### Experimental Groups

Thirty-six Wistar-albino female rats taken from Selçuk University Experimental Medicine Application and Research Center were divided into five groups.

**Fig. 1:**
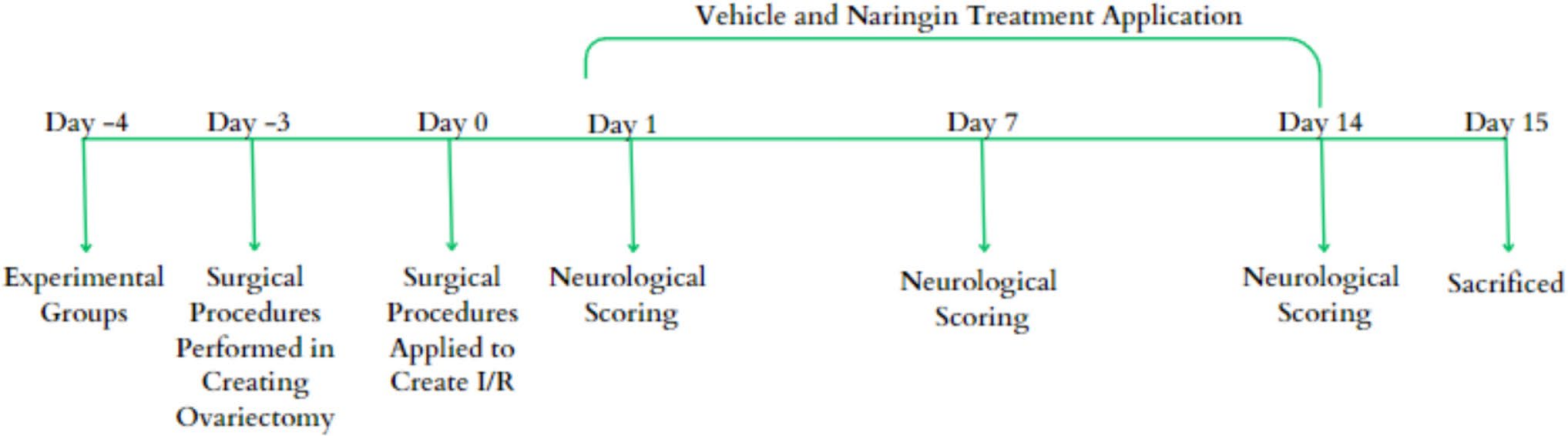
Study protocol

1. Control group (*n* = 6): No anesthesia or surgical procedures were performed on the animals in this group. Neurological scoring was performed on the 1 st, 7 th, and 14 th days after brain I/R surgery in the other groups. After the 14 th day, the rats were sacrificed by cervical dislocation.
2. Ovariectomy-sham brain I/R group (*n* = 6): After general anesthesia was established for the animals in this group, sham I/R was achieved by opening and closing the carotid artery regions. Neurological scoring was performed on the 1 st, 7 th, and 14 th days after brain I/R surgery. The animals were not subjected to any treatment. After the 14 th day, the rats were sacrificed by cervical dislocation.
3. Ovariectomy-I/R group (*n* = 8): Three days after the ovariectomy procedure was performed under general anesthesia, carotid arteries were isolated and ligated, and ischemia was performed in the rats under general anesthesia. Neurological scoring was performed on the 1 st, 7 th, and 14 th days after brain I/R surgery. After the 14 th day, the rats were sacrificed by cervical dislocation.
4. Ovariectomy-I/R sham treatment group (*n* = 8): After ovariectomy was performed under general anesthesia, the carotid arteries were isolated and ligated, and ischemia was performed in the rats under general anesthesia. Vehicle application was made for 14 days after ischemia. Neurological scoring was performed on the 1 st, 7 th, and 14 th days after brain I/R surgery. After the 14 th day of vehicle application, the rats were sacrificed by cervical dislocation.
5. Ovariectomy-I/R naringin treatment group (*n* = 8): After ovariectomy was performed under general anesthesia, the carotid arteries were isolated and ligated, and ischemia was performed in the rats under general anesthesia. Figure 1 indicates study protocol. Naringin was administered for 14 days after ischemia (Fig. 1). Neurological scoring was performed on the 1 st, 7 th, and 14 th days after brain I/R surgery. After the 14 th day of naringin treatment, the rats were sacrificed by cervical dislocation.

### Surgical Procedures Performed in Creating Ovariectomy

Before the surgical procedure, general anesthesia was achieved by intraperitoneal administration of a mixture of ketamine HCl 60 mg/kg and xylazine HCl 5 mg/kg. Following anesthesia, the abdominal area was shaved and the abdomen was entered through an incision. The fallopian tubes were ligated bilaterally with silk thread, and the ovaries were removed. The area was closed, and the wound was waited for healing.

### Surgical Procedures Applied to Create I/R

Three days after the ovariectomy procedure, general anesthesia was achieved by intraperitoneal administration of a mixture of ketamine HCl 60 mg/kg and xylazine HCl 5 mg/kg to the sham ischemia–reperfusion and I/R groups.

The neck area of the rats in the I/R groups was shaved after anesthesia. A ventral incision was made in the midline of the neck. The right and left common carotid arteries were isolated from the surrounding tissues and revealed. Bilateral common carotid artery occlusion was performed, and ischemia was created for 30 min and kept at 25 °C. After 30 min of ischemia, reperfusion was achieved, and the incision was stitched.

The neck area of the rats in the sham I/R group was shaved after anesthesia. A cervical incision was made in the ventral midline of the neck, the right and left common carotid arteries were isolated, and the incision was sutured without ligation [28–30].

### Neurological Evaluation

Following I/R, neurological scoring was performed on the 1 st, 7 th, and 14 th days of naringin treatment to determine the severity of ischemia–reperfusion-induced damage and to evaluate the effect of the treatment on the motor functions of the rats. In the neurological evaluation, the rats were lifted into the air by their tails, and the flexing of the forelimbs was observed. Normally, the front legs of rats extend toward the ground. In contrast, in infarcted rats, shoulder adduction with wrist flexion and elbow extension, adduction with wrist and elbow flexion, and shoulder internal rotation can be observed [31]. It was then placed on towel paper, and its ability to resist was evaluated by applying lateral pressure while holding its tail. While normal and mild dysfunction resist pressure, rats with severe dysfunction have low resistance [31]. Then, the rats’ walking and circling behavior while walking was evaluated (Table 1). Table 1 indicates neurological scoring.

**Table 1:** Neurological scoring (Bederson ve ark 1986)

Score	Findings
0	No functional defects
1	Variable degrees of forefoot flexion
2	Variable degrees of forefoot flexion Minimal or no resistance to lateral pressure
3	Variable degrees of forefoot flexion Minimal or no resistance to lateral pressure Creating a circle while walking

### Real-Time Quantitative Polymerase Chain Reaction (qPCR) Analysis

Real-time qPCR analysis was applied to determine the expression of the calbindin, alpha tubulin, and beta tubulin genes.

### RNA Isolation from the Hippocampus and Frontal Cortex

To determine the expression of the NeuN gene by real-time qPCR analysis, total RNA was first isolated using a commercial kit (Bio Basic; Canada). Hippocampus and frontal cortex tissues were weighed, and 25–50-mg samples were taken. One millimeter of lysis buffer (miRNA extractor) was added to the samples, and they were lysed on ice with a homogenizer. Homogenized samples were kept at room temperature for 5–10 min. Then, 0.2 ml (200 µl) chloroform was added to the samples and vortexed for 30 s. After vortexing, samples were centrifuged at 12,000 × g for 10 min at 4 °C. As a result of centrifugation, the samples were divided into three: organic phase, middle phase, and upper phase. The upper phase containing RNA was taken and transferred to a 1.5-ml RNase-free tube, and 1.5 times its volume of 100% ethanol was added and mixed by pipetting. The mixture was transferred to the spin column and centrifuged at 12,000 × g for 2 min. Then, 0.5 ml of RPE solution was added to the spin column and centrifuged repeatedly at 12,000 × g for 30 s. After this step was repeated several times, the column was placed in a new centrifuge tube, and 50 µl of RNase-free water was added and kept at room temperature for 2 min. After waiting, it was centrifuged for the last time at 12,000 × g for 30 s.

Nanodrop SMA 1000 (Merinton, China) was used to determine the amount and purity of the RNAs obtained as a result of the analysis. The OD260/280 ratio was calculated according to their optical density, and RNAs with values above 1.9 were used in the study. To use in cDNA synthesis, the obtained total RNAs were stored at − 20 °C.

### cDNA Synthesis

The mRNA in the total RNA obtained as a result of RNA isolation was converted into cDNA using a commercial kit (OneScript Plus, ABM; Canada). Equal amounts of samples were taken, and the protocol recommended by the manufacturer prepared a cDNA synthesis mixture.

For each sample, 10 μL of the mixture was added onto 10 μL of RNA sample and mixed by pipetting. The tubes were placed in the Thermal Cycler (Bio-Rad, USA) device, and the reverse transcription program was run with the protocol specified in the kit. In the first step of the program, cDNA synthesis was performed at 55 °C for 15 min, then denaturation was performed at 85 °C for 5 min, and in the last step, it was kept at + 4 °C. cDNA amounts and purity were measured with nanodrop. Samples were stored at − 20 °C until qPCR was performed (Tables 2, 3, and 4). Table 2 shows the cDNA synthesis mixture prepared to obtain cDNA from total RNA. Table 3 presents the primers used for real-time PCR. Table 4 indicates the reaction content for PCR.

**Table 2:** cDNA synthesis mixture prepared to obtain cDNA from total RNA

Component	Quantity
5 × RT buffer	4 μL
dNTP	1 μL
Primers	1 μL
OneScript Plus RTase	1 μL
Nuclease free H20	3 μL

**Table 3:** Primers used for real-time PCR

Gene	Primer sequence	Function/accession number
Kalbindin F	5′-ATTTCGACGCTGACGGAAGT-3′	Target gene NM_031984.2
Kalbindin R	5′-AGGTGATAACTCCAATCCAGCC-3′	Target gene NM_031984.2
Beta tubulin F	5′-GCAACCAGATCGGGGCCA-3′	Target Gene NM_139254.2
Beta tubulin R	5′-GTTCATGATGCGGTCGGGAT-3′	Target gene NM_139254.2
Alfa tubulin F	5′-GGAGCTCTACTGCCTGGAACAT-3′	Target gene XM_063264205.1
Alfa tubulin R	5′-CAATAACTGTGGGTTCCAGGTCTAC-3′	Target gene XM_063264205.1
GAPDH F	5′-GGGCCAAAAGGGTCATCATC-3′	Referance gene NM_017008.4
GAPDH R	5′-AACCTGGTCCTCAGTGTAGC-3′	Referance gene NM_017008.4

**Table 4:** Reaction content for PCR

Component	Quantity
Master mix (enzyme, dNTP, Mg, buffer, and water)	10 μl
cDNA	5 μl
Further primer	1 μl
Back primer	1 μl
Nuclease-free H_2_O	3 μl

### Determination of Expression of Calbindin, Alpha Tubulin, and Beta Tubulin Genes

cDNA samples obtained from the hippocampus and frontal cortex tissues of control group rats without any intervention were used and diluted according to the ratios of 1/1, 1/2, 1/4, 1/8, 1/16, and 1/32. For the primers to bind at the optimum temperature, temperature ranges were tested and standardization was made.

Primers used for analysis of target gene expression at the mRNA level were obtained from the manufacturer (Sentebiolab, Turkey). GAPDH synthesized by Oligomer (Turkey) was used as the reference gene. Approximately 50 ng of primer was used in each reaction (Table 4).

PCR reactions using a ready-made commercial kit (BlasTaq 2X qPCR Master Mix, ABM, Canada) were performed in a total volume of 20 μl. The components used for the reaction were prepared according to the amounts in the table.

After optimization of the primers, PCR conditions were adjusted. Enzyme activation was carried out at 95 °C for 3 min, denaturation at 95 °C for 10 s, and binding and polymerization in 40 cycles for 1 min at 60 °C. The reactions were carried out using the CFX Connect Real-Time PCR Detection System (Bio-Rad, USA), and the analysis results were evaluated using the 2^−ΔΔCT^ method developed by Livak and Schmittgen [32].

### Immunohistochemical Studies

Thirty-six hippocampus and frontal cortex samples taken from rats were kept in cooled 4% paraformaldehyde fixative at + 4 °C for 24 h with a fixative/tissue volume ratio of 10/1. Tissues were prepared for the frozen section by placing them in 30% sucrose and waiting until the tissue settled to the bottom. The desired section surface was angled and embedded in the cryomatrix. Serial sections of a 5-μm thickness were taken with the Thermo Shandon Cryostat 210160 GB on the cryostat device. The sections were placed in the freezer to maintain − 20 °C in the frozen device.

For immunofluorescence staining, the sections were removed from the freezer and applied first with PBS and then with Triton × 100 in a humidity box for 10 min. Then, they were washed with PBS three times for 5 min. They were incubated with the block solution at room temperature for 30 min, and the remaining excess liquid was aspirated.

### Immunohistochemical Staining: Anti-NeuN Antibody Labeling

Anti-NeuN antibody labeling protocol:

1. The sections were marked around the sections with a hydrophobic pen.
2. It was treated with anti-NeuN (ab276297) (1/100 diluted) antibody for 24 h at + 4 °C.
3. Washed with PBS three times for 5 min each.
4. Incubated with DyLight 550 (ab69888) (1/100 diluted) antibody for 2 h at room temperature in the dark.
5. The sections were covered with a DAPI fluorescent covering medium (Fluoroshield with DAPI, F6057, Sigma Aldrich, USA).

Frontal cortex and hippocampus frozen sections were labeled with anti-NeuN antibody (ab279297) and goat anti-ay DyLight 550 (abcam; ab96888) as the secondary antibody. The procedures were carried out in a dark environment by creating a humidity box. Examinations were made on an Olympus BX51 Trinocular fluorescence microscope. Four areas were randomly selected from each of the determined areas and recorded with a DP72 camera at 40 × magnification. Field verification was performed with DAPI nucleus stain. The ratio of anti-NeuN labeled cells to total cells in the recorded images was calculated using the ImageJ (National Institutes of Health, Bethesda, MD, USA) program.

### Statistics

The suitability of the quantitative data in the groups to normal distribution was examined with the single-sample Kolmogorov–Smirnov test. Variables that did not comply with normal distribution were transformed into a normal distribution, and multivariate analysis of variance (MANOVA) was used to compare quantitative values for groups at different periods. In the study, the *P* < 0.05 value was accepted as the limit of statistical significance.

## Results

### Neurological Scoring Results

**Fig. 2:**
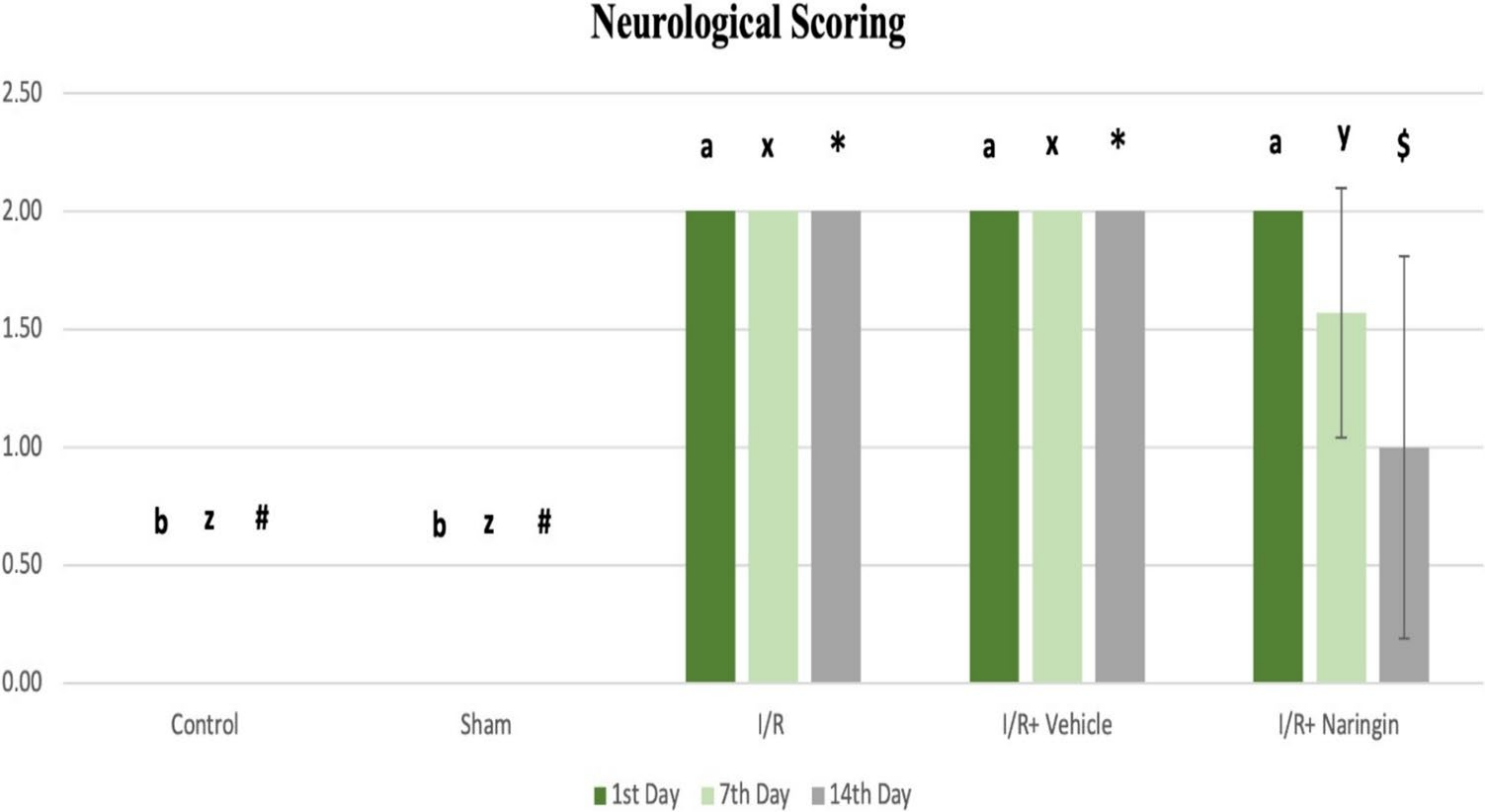
Neurological scoring between groups (a > b), x > y; * > $ > #. I/R lead to increased neurological scores. Naringin treatment, however, reduced neurological scores

The differences in neurological recovery in animals within and between groups are shown in the table and figure below. In the intra-group evaluation, the score is 0 because no treatment was performed on the control group. Since sham ischemia was applied in the sham I/R group, there was no damage in neurological scoring as in the control group. In the ischemia–reperfusion and sham treatment group, the score was determined to be 2 due to the effect of ischemia. In the treatment group, while the score was 2 at the first evaluation following ischemia, with the effect of the treatment, the score was determined to be 1.57 on day 7, and then the score was determined to be 1 as a positive effect of the 14-day treatment. According to the neurological scoring results, the control, sham I/R, I/R, and sham treatment groups did not show any changes on days 1, 7, and 14. However, the neurological score decreased in the treatment group (Fig. 2; Table 5). Figure 2 indicates neurological scoring between groups. Table 5 presents neurological scoring findings.

**Table 5:** Neurological scoring findings

Groups	**1 day (mean ± SD)**	**7 days (mean ± SD)**	**14 days (mean ± SD)**
Control group	0.00 ± 0.00 c	0.00 ± 0.00 c	0.00 ± 0.00 c
Sham group	0.00 ± 0.00 c	0.00 ± 0.00 c	0.00 ± 0.00 c
I/R group	2.00 ± 0.00 a	2.00 ± 0.00 a	2.00 ± 0.00 a
I/R + vehicle group	2.00 ± 0.00 a	2.00 ± 0.00 a	2.00 ± 0.00 a
I/R + naringin group	2.00 ± 0.00 a	1.57 ± 0.53 b	1.00 ± 0.81 b

Letters in the same column show statistical differences (a > b > c >) (*p* < 0.001)

### Calbindin Gene Expression Results

**Fig. 3:**
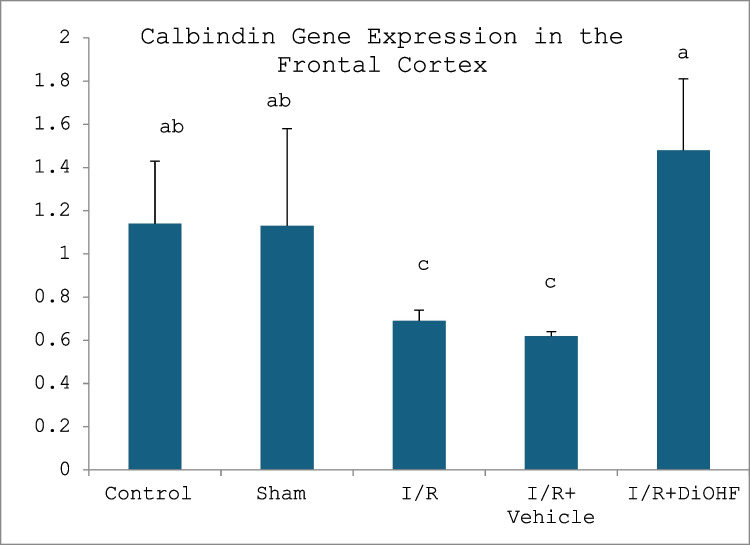
Expression level of the calbindin gene in the frontal cortex in the experimental groups. Expression of the calbindin gene did not change in the control and sham groups. It decreased in the I/R and I/R + vehicle groups. A significant increase was seen in the I/R + naringin group, to which Naringin supplementation was applied (a > ab > c; *P* < 0.01)

**Fig. 4:**
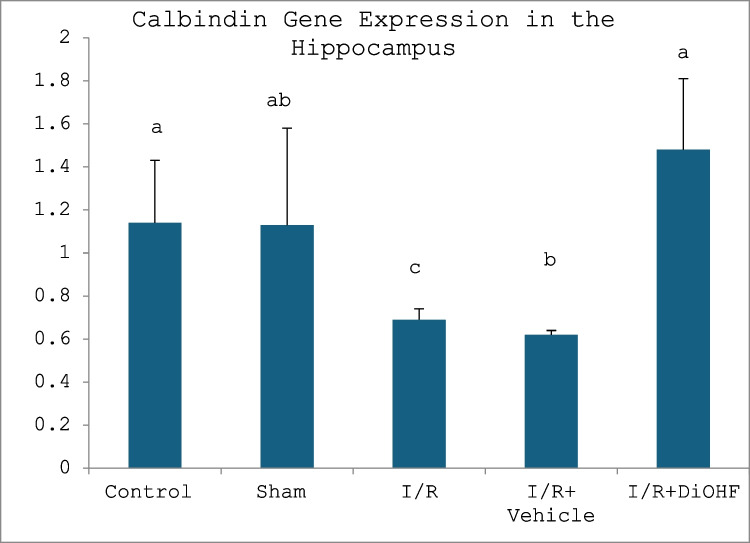
Expression level of the calbindin gene in the hippocampus in the experimental groups. Expression of the calbindin gene did not change in the control and sham groups. It decreased in the I/R and I/R + vehicle groups. A significant increase was seen in the I/R + naringin group (a > ab > c; *P* < 0.01)

The expression level of the calbindin gene in frontal cortex and hippocampus tissues is given in the Tables 6 and 7 and Figs. 3 and 4. Figure 3 presents the expression level of the calbindin gene in the frontal cortex in the experimental groups. Figure 4 indicates the expression level of the calbindin gene in the hippocampus in the experimental groups. Tables 6 and 7 present frontal cortex and hippocampus calbindin levels.

**Table 6:** Calbindin gene expression in the hippocampus

Groups	**(Mean ± SD)**
Control group	0.94 ± 0.26 ab
Sham group	0.92 ± 0.42 ab
I/R group	0.51 ± 0.29 c
I/R + vehicle group	0.71 ± 0.12 c
I/R + naringin group	1.33 ± 0.27 a

Letters in the same column show statistical differences a > b > c (*p* < 0.01)

**Table 7:** Expression level of calbindin gene in frontal cortex

Groups	**(Mean ± SD)**
Control group	1.14 ± 0.29 ab
Sham group	1.13 ± 0.45 ab
I/R group	0.69 ± 0.05 c
I/R + vehicle group	0.62 ± 0.02 c
I/R + naringin group	1.48 ± 0.33 a

Letters in the same column show statistical differences (a > ab > c; *P* < 0.01)

According to data obtained from the hippocampus, calbindin gene expression did not show any difference between the control and sham groups. A decrease in calbindin gene expression was observed in the I/R and I/R + vehicle groups. In the I/R + naringin group, in which naringin supplementation was applied, this led to a significant increase in neurogenesis.

According to the data obtained from the frontal cortex, calbindin gene expression is at similar levels in the control and sham groups. While the expression level decreased in the I/R and I/R + vehicle groups, the decrease in the expression level was prevented due to the naringin supplementation applied to the I/R + naringin group.

#### Alpha Tubulin Gene Expression Results

**Fig. 5:**
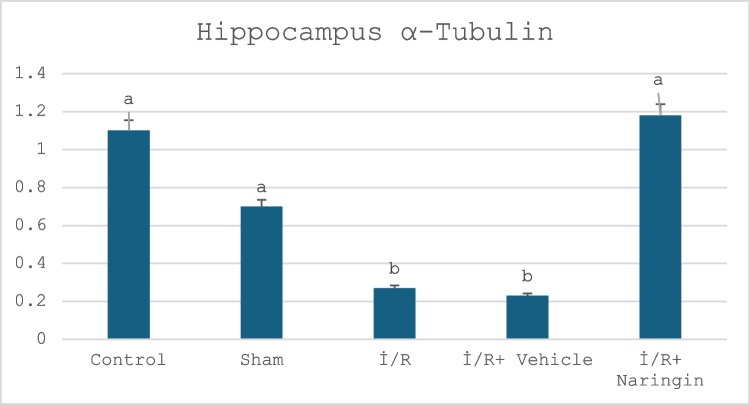
The expression level of the α-tubulin gene in the hippocampus. α-tubulin expression was at similar levels in the control and sham groups but decreased in the I/R and I/R + vehicle groups. In the I/R + naringin group, it improved again and rose to the level of the control and sham groups

**Fig. 6:**
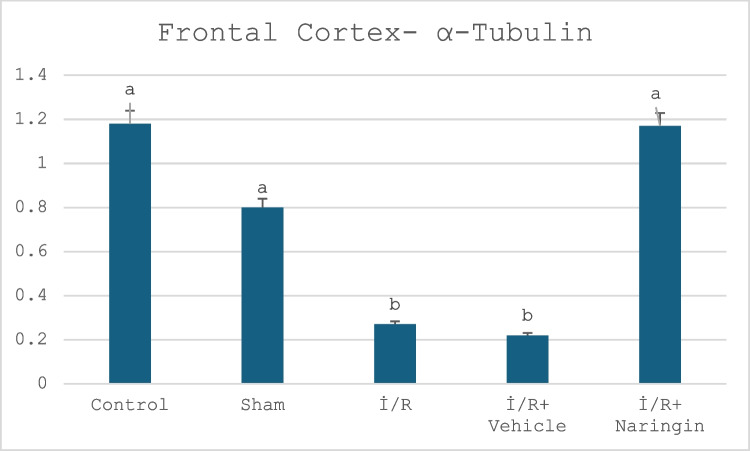
Expression level of α-tubulin gene in frontal cortex. α-Tubulin expression was at similar levels in the control and sham groups, but decreased in the I/R and I/R + vehicle groups. In the I/R + naringin group, it increased with the effect of the treatment and reached the level of the control and sham groups

The expression level of the alpha-tubulin gene in the hippocampus and frontal tissues is given in Tables 8 and 9 and Figs. 5 and 6. Figure 5 presents the expression level of the alpha-tubulin gene in the frontal cortex in the experimental groups. Figure 6 indicates the expression level of the alpha-tubulin gene in the hippocampus in the experimental groups. Tables 8 and 9 present the frontal cortex and hippocampus alpha tubulin.

**Table 8:** The expression level of the alpha tubulin gene in the hippocampus

Groups	**(Mean ± SD)**
Control group	1.10 ± 0.46 a
Sham group	0.70 ± 0.14 a
I/R group	0.27 ± 0.03 b
I/R + vehicle group	0.23 ± 0.15 b
I/R + naringin group	1.18 ± 0.13 a

Letters in the same column show statistical differences (a > b > c; *P* < 0.01)

**Table 9:** The expression level of the alpha tubulin gene in the frontal cortex

Groups	**(Mean ± SD)**
Control group	1.18 ± 0.29 a
Sham group	0.80 ± 0.19 a
I/R group	0.27 ± 0.01 b
I/R + vehicle group	0.22 ± 0.06 b
I/R + naringin group	1.17 ± 0.05 a

Letters in the same column show statistical differences (a > b > c; *P* < 0.01)

Data obtained from the hippocampus showed that alpha tubulin gene expression did not change for the control and sham groups and was at similar levels. Alpha tubulin expression decreased in the I/R and I/R + vehicle groups. I/R caused a reduction in alpha tubulin; however, naringin supplementation after I/R led to an increase in tubulin levels (group 5). According to the data obtained in the frontal cortex, alpha tubulin expression showed no difference in the control and sham groups. In the I/R and I/R + vehicle groups, a decrease in expression levels was observed due to the effect of I/R. Naringin treatment had a positive effect, increasing the expression level of the I/R + naringin group and reaching the level of the control group.

### Beta Tubulin Gene Expression Results

**Fig. 7:**
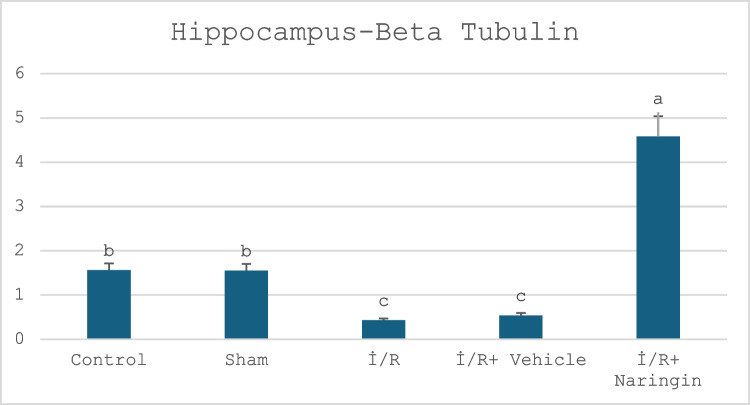
Expression level of beta tubulin gene in the hippocampus in experimental groups. The expression levels of the beta tubulin gene did not change in the control and sham groups, but a decrease was observed in the I/R and I/R + vehicle groups due to the I/R effect. The group treated with naringin showed an increase, exceeding the level of the control group (a > b > c; *P* < 0.01)

**Fig. 8:**
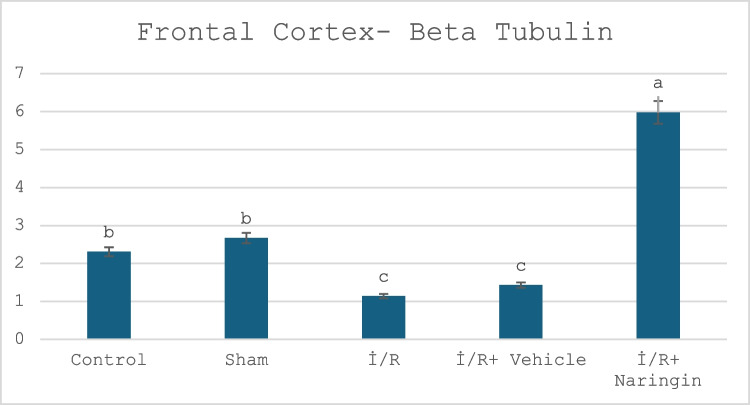
Expression levels of beta tubulin gene in frontal cortex in experimental groups. Expression levels of the beta-tubulin gene did not change in the control and sham groups. It decreased in the I/R and I/R + vehicle groups. In the naringin treatment group, the expression level increased with the effect of the treatment, exceeding the levels of the control and sham groups (a > b > c; *P* < 0.01)

The expression levels of the beta tubulin gene in the hippocampus and frontal cortex are given in Tables 10 and 11 and Figs. 7 and 8. Figure 7 presents the expression level of the beta tubulin gene in hippocampus in the experimental groups. Figure 8 indicates the expression level of the beta tubulin gene in the frontal cortex in the experimental groups. Tables 10 and 11 present the frontal cortex and hippocampus beta tubulin.

**Table 10:** Beta tubulin expression in the hippocampus

Gruplar	(Mean ± SD)
Groups	1.56 ± 0.24 b
Control group	1.55 ± 0.33 b
Sham group	0.43 ± 0.15 c
I/R group	0.54 ± 0.35 c
I/R + vehicle group	4.50 ± 0.63 a

Letters in the same column show statistical differences (a > b > c; *P* < 0.01)

**Table 11:** Beta tubulin expression in the frontal cortex

Groups	**(Mean ± SD)**
Control group	2.31 ± 0.31 b
Sham group	2.67 ± 0.36 b
I/R group	1.14 ± 0.14 c
I/R + vehicle group	1.43 ± 0.26 c
I/R + naringin group	5.98 ± 1.85 a

Letters in the same column show statistical differences (a > b > c; *P* < 0.01)

According to hippocampus data, beta tubulin gene expression shows no difference in the control and sham groups. Beta tubulin expression level decreased in the I/R and I/R + vehicle groups. In the I/R + naringin group, beta tubulin expression level increased.

According to frontal cortex data, beta tubulin gene expression showed no difference between the control group and the sham group. Beta tubulin gene expression decreased in the I/R and I/R + vehicle groups compared to the control and sham groups due to I/R damage. In the I/R + naringin group, beta tubulin expression increased with the effect of the treatment.

### Anti-NeuN Antibody Labeling Results

**Fig. 9:**
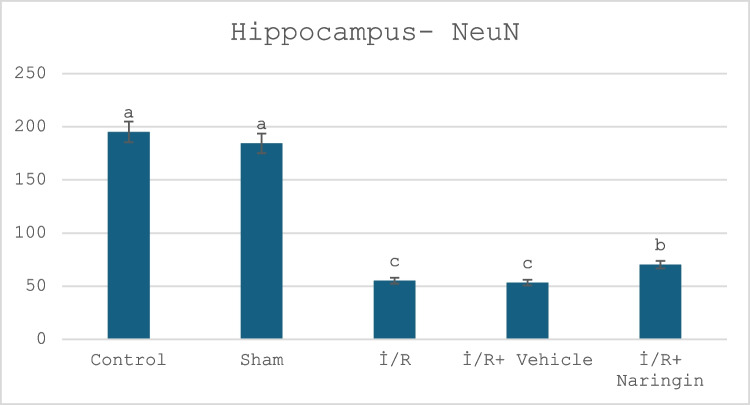
Levels of anti-NeuN antibody in the hippocampus in the experimental groups. The number of cells stained with NeuN did not change in the control and sham groups. It decreased in the I/R and I/R + vehicle groups and increased in the I/R + naringin group, but could not reach the level of the control and sham groups (a > b > c; *P* < 0.001)

**Fig. 10:**
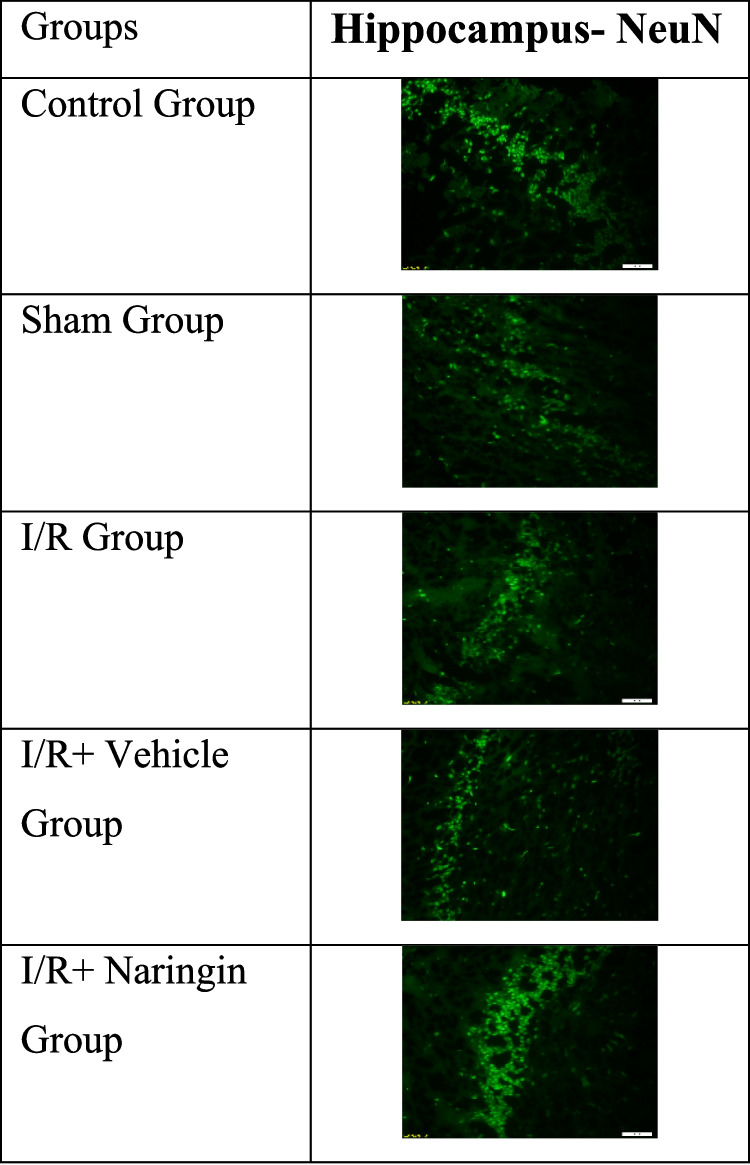
Immunofluorescent labeling of NeuN in the hippocampus

**Fig. 11:**
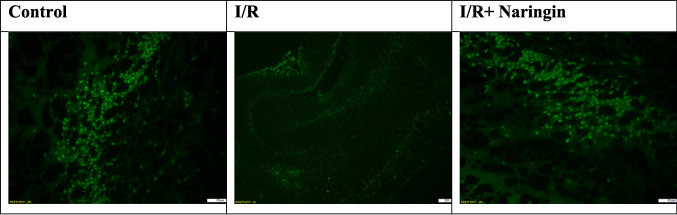
Immunofluorescence labeling of the hippocampus

**Fig. 12:**
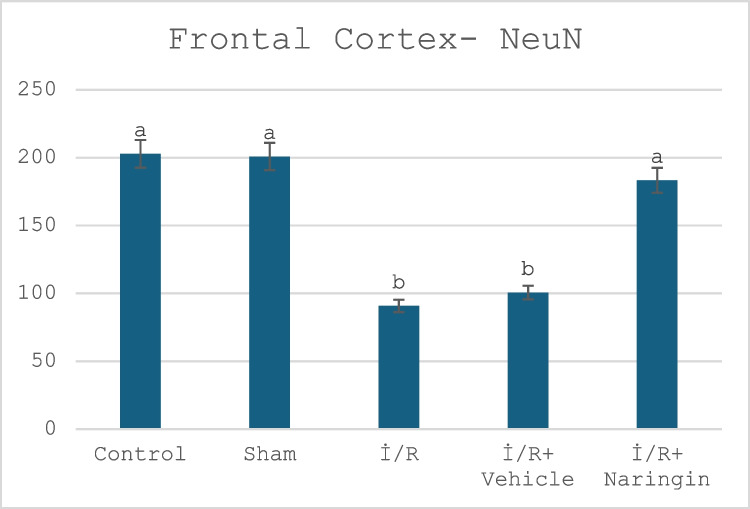
Level of anti-NeuN antibody in the frontal cortex in experimental groups. The number of cells stained with NeuN did not change in the control and sham groups, but decreased in the I/R and I/R + vehicle groups, and increased with the effect of the treatment in the I/R + naringin group, reaching the levels of the control and sham groups (a > b >; *P* < 0.001)

**Fig. 13:**
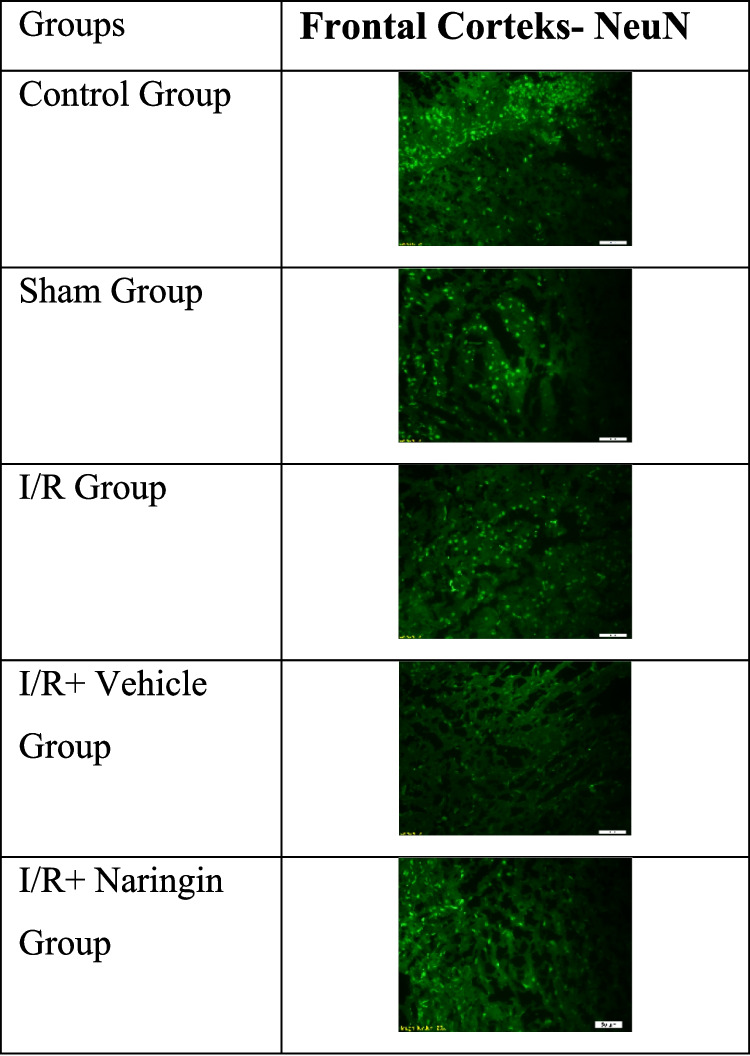
Fluorescent images of NeuN with immune marker in frontal cortex

The expression levels of the anti-NeuN antibody, which is expressed by mature neurons, in the hippocampus and frontal cortex tissues are given in Tables 12 and 13 and Figs. 9, 10, 11, 12, and 13. Table 12 indicates the level of the anti-NeuN antibody in the hippocampus. Table 13 presents the level of the anti-NeuN antibody in the frontal cortex. Figure 9 shows the levels of the anti-NeuN antibody in the hippocampus in the experimental groups. Figure 10 indicates immunofluorescent labeling of NeuN in the hippocampus. Figure 11 presents immunofluorescence labeling of the hippocampus. Figure 12 indicates the level of the anti-NeuN antibody in the frontal cortex in the experimental groups. Figure 13 presents fluorescent images of NeuN with the immune marker in the frontal cortex.

**Table 12:** Level of anti-NeuN antibody in the hippocampus

Groups	**(Mean ± SD)**
Control group	195.18 ± 30.08 a
Sham group	184.41 ± 30.67 a
I/R group	55.25 ± 17.40 c
I/R + vehicle group	53.37 ± 22.30 c
I/R + naringin group	70.34 ± 20.12 b

Letters in the same column show statistical differences (a > b > c; *P* < 0.001)

**Table 13:** Level of anti-NeuN antibody in the frontal cortex

Groups	**(Mean ± SD)**
Control group	202.88 ± 30.83 a
Sham group	200.88 ± 30.71 a
I/R group	90.76 ± 41.23 b
I/R + vehicle group	100.63 ± 31.88 b
I/R + naringin group	183.34 ± 30.70 a

Letters in the same column show statistical differences (a > b > c; *P* < 0.001)

When the anti-NeuN antibody level is examined according to the data obtained from the hippocampus, the anti-NeuN antibody levels of the control and sham groups are close. Anti-NeuN antibody levels decreased in the I/R and I/R + vehicle groups. An increase was observed in the I/R + naringin group, but it did not reach the levels of the control and sham groups.

According to study data, anti-NeuN antibody levels in the frontal cortex are at similar levels in the control and sham groups. Anti-NeuN antibody expression decreased in the I/R and I/R + vehicle groups. In the I/R + Naringin group, an increase in the anti-NeuN antibody level was observed due to the effect of the treatment, and it increased to the level of the control and sham groups.

## Discussion

When the findings of the study were evaluated in general, it was determined that neurological failure occurred due to brain ischemia reperfusion and that factors such as calbindin, tubulin, and Neu N, which lead to neurogenesis, showed a significant decrease. However, it was observed that naringin application for 2 weeks after brain I/R significantly improved the changes caused by I/R. We suggest that neurogenesis-related genes are increased in neuronal stem cells in the hippocampus to compensate for ischaemia–reperfusion injury due to naringin treatment. These stem cells then migrate to damaged areas such as the frontal cortex and play a role in repairing the damage.

The first finding evaluated in the study was the examination of neurological failure as a result of I/R and the effectiveness of the treatment. It was determined that neurological score values increased significantly with the effect of I/R, but showed a significant improvement depending on the treatment and its duration.

There are many studies on the effect of flavonoids on neurological recovery after I/R. Studies show that flavonoid treatment applied after ischemia improves the high neurological score caused by I/R. In the study conducted by Huang et al., the effectiveness of Nobiletin, an active natural flavonoid component of citrus fruits, on cerebral (I/R) damage was investigated. The nobiletin high-dose group (20 mg/kg) and nobiletin low-dose group (10 mg/kg) were created, and intragastric administration was performed for 7 days following ischemia reperfusion. According to the neurological evaluation score made after the application, while the score was significantly higher in the I/R group, it was significantly lower in the nobiletin-treated group. However, there was no significant difference between dose groups [33]. In the study conducted by Guo et al. to elucidate the neuroprotection mechanism of quercetin, which has a basic neuroprotective effect on experimental ischemic stroke, against cerebral I/R injury, it was shown that quercetin alleviates I/R injury and improves the neurological score [34].

The effect of orientin, a flavonoid monomer obtained from *Cyperus esculentus* L. leaves, on neurological scoring was observed by applying different doses to experimental groups. Five hundred milligrams per kilogram were administered to the *C. esculentus* L. high-dose group with 30% elution, 125 mg/kg was administered to the *C. esculentus* L. low-dose group with 30% elution, and distilled water was administered intragastrically to the model group for 7 days. Neurological scoring after treatment showed that orientin flavonoid could alleviate neurological dysfunction, but there was no different dose-dependent effect [35].

Breviscapine, the most commonly used crude extract from plants clinically, was administered for 30 days at high (60 mg/kg), medium (30 mg/kg), and low (15 mg/kg) doses. Neurological function was scored with the Bederson score table. According to the scoring, the low dose reduced the neurological score less, while as the dose increased, the neurological score decreased further; however, no significant difference was found between them [36].

A study on the pharmacological effects of fisetin on protein damage during cerebral I/R injury used a modified neurological severity score to examine neurological disorders. It has been shown that fisetin administration significantly alleviates neurological deficits caused by middle cerebral artery occlusion followed by reperfusion (MCAO/R) [37]. Another study investigated the healing effect of luteolin-7-O-β-d-glucuronide (LGU) on cerebral ischemic injury. It is the first time that LGU has been proven to protect against cerebral ischemia in an MCAO model with an improved neurological deficit score. In the study conducted by Janyou et al., it was reported that the application of isoquercetin at doses of 10 and 20 mg/kg significantly reduced neurological damage due to ischemia–reperfusion [38]. In another study, the application of lithium together with rutin, a flavonoid, significantly improved the neurological score after cerebral I/R in rats [39]. In their study, Zhang et al. reported that kaempferol, a natural plant flavonoid, significantly suppressed the neurological score severity [40].

In our study, the increased neurological score values were suppressed in female rats by intraperitoneal administration of naringin at a dose of 100 mg/kg, first with ovariectomy and then with 30 min of ischemia and 2 weeks of reperfusion. Another parameter examined in the current study was calbindin. While the value of this parameter decreases with I/R in both the hippocampus and frontal cortex, it increases with a 2-week naringin treatment. Calbindin is a cytosolic bim-binding protein that protects neurons from harmful factors [17]. Calbindin, which binds to cellular structures in neurons, is an important protein that plays a role in the regulation of calcium pools, which are important for synaptic plasticity [18]. It prevents neuron death by inhibiting different pro-apoptotic pathways [19]. Although it is thought to have neuroprotective functions, this view is not generally supported [41]. Although there are studies showing that calbindin levels do not decrease with age, there are also studies showing that it has no relationship with age [17]. In a study conducted, alpha syn aggregation, calbindin expression, and the interaction between oxidative stress were investigated by examining calbindin-positive neurons and alpha syn involvement in Lewy body dementia and normal cases, as well as in a unilateral rotenone mouse model. In this study, increased calbindin + neurons were found in Lewy body dementia cases compared to normal cases [42]. When investigating the long-term effects of soybean isoflavones on changes in calbindin immunoreactivity in the hippocampus in middle-aged ovariectomized female rats, as well as middle-aged control female and male rats, calbindin immunoreactivity in the hippocampal Cornu Ammonis 1 (CA1) region in ovariectomized females was similar to that in control females, while calbindin immunoreactivity in males was significantly lower than that in control females. In the dentate gyrus (DG), calbindin immunoreactivity in ovariectomized females and males was significantly lower than that in control females. Calbindin immunoreactivity was determined to increase dose-dependently after isoflavone treatment in the CA1 region and DG [43]. Multiplex immunostaining data for calycosin, an important isoflavonoid compound in *Astragalus membranaceus*, showed that knockdown of Ugt1 caused a significant reduction in calbindin-positive neurons and NeuN + cells compared to the control group. Immunohistochemistry showed that the number of calbindin-positive neurons and NeuN + cells increased, and DNA damage decreased after calycosin treatment in Ugt1 knockout mice [44]. Another study showed that the flavonoid lysium could improve irradiation-induced animal weight loss, depressive behavior, spatial memory impairment, and hippocampal neuron loss. An immunohistochemistry study showed that irradiation-induced damage was significantly improved by lysine treatment [34]. Quercetin supplementation ameliorated cerebellar tissue apoptosis and gliosis, and improved calbindin levels. Calbindin immunostaining showed intense cytoplasmic immune reaction in many patients [45]. Persistent changes in hippocampal expression of calbindin may contribute to memory impairments after neonatal hypoxia–ischemia, as persistent Ca 2 + dysregulation after hypoxia–ischemia (HI) may lead to ongoing injury. As a result, neonatal HI causes a long-term impairment in calbindin increase in the CA subfields of the hippocampus at a critical stage of development [46].

Hippocampal dentate granule cells normally express the calcium-binding protein calbindin and are the hippocampal neurons least sensitive to ischemic insult in adults. The main finding in this study is that a subset of hippocampal dentate granule cells is highly vulnerable to hypoxia/ischemia only during development, and that sensitive granule cells are particularly those that are least mature and do not yet express the putatively protective calcium-binding protein calbindin. The results show that there is a correlation between the development of resistance to ischemic damage and the development of calbindin expression during the cell maturation process [47]. While cerebral ischemia significantly increases infarct volume, MCAO injury causes a decrease in calbindin expression. Both in vivo and in vitro results showed a decrease in calbindin after neuronal cell damage. These results indicate that decreased calbindin in ischemic brain injury contributes to neuronal cell death. These findings indicate that calbindin reductions cause imbalances in calcium homeostasis, which leads to neuronal cell death in MCAO-induced damage and glutamate-exposed damage [48]. In a study, application of quercetin flavonoid led to observable changes associated with an increase in calbindin level [45]. It alleviated cerebral I/R-induced neurological disorders, brain infarction, disruption of the blood–brain barrier, and oxidative stress [49]. In our study, similar to the studies mentioned above, naringin supplementation for 2 weeks significantly increased the levels of calbindin suppressed by I/R. When the existing literature is examined, it is seen that there is no research examining the I/R-naringin-calbindin relationship, which can be considered the first research finding on this subject. As another finding in our study, tubulin values were examined in the frontal cortex and hippocampus, and this parameter decreased with ischemia–reperfusion and increased again with blood naringin treatment. Microtubules, composed of alpha and beta tubulin heterodimers, play vital roles in numerous cellular processes, including intracellular traffic, migration, and mitosis. Alpha and beta tubulins are two highly conserved proteins within the tubulin superfamily [12]. Alpha I tubulin (TUBA1 A) is generally not found in the proliferative ventricular zone during development [13]. In adulthood, it is expressed in various neural structures and in the subgranular zone (SGZ) and subventricular zone (SVZ) [14]. While TUBA1 A is expressed in type-4 and type-5 cells, it is not expressed in type-1, type-2, and type-3 cells [13].

Tubulin beta 3 class III (TUBB3) expression is associated with the earliest stages of neuronal differentiation; it is at a high level in the cortex in the first postnatal period and decreases over time with increasing postnatal development [16]. In the model created to investigate the effect of troxerutin flavonoid, which has neuroprotective properties, on increasing neurite growth and migration of NSCs from the SVZ, the branching amount of TUBB3 neurons was examined. Compared with the control group, the troxerutin flavonoid–treated group exhibited longer neurites with more branching [50]. The effects of the flavonoid casticine extracted from *Croton betulaster* on rat cerebral cortex neurons were examined in vitro, showing that treatment of neural progenitors with 10 μM casticin increased the population of neurons positive for the neuronal marker TUBB3 and neuronal transcriptional factor Tbr2 by approximately 20%, as well as a 50% reduction in neuron death that has been observed [51]. One study determined that fisetin, a plant-derived polyphenol compound, was able to inhibit the aggregation of tau fragment K18 and cleave tau K18 filaments in vitro. However, it has been determined that fisetin does not affect tau-induced tubulin assembly, including kinetics or final optical absorbance, which is an indicator of the amounts of microtubules formed [52].

Treatment with apigenin was observed to preserve neuron and astrocyte integrity, as determined by Rosenfeld staining and immunocytochemistry for TUBB3 and glial fibrillary acidic protein (GFAP), respectively. Additionally, it was observed by Fluoro-Jade B and caspase 3 immunostaining that apigenin was not neurotoxic and had a neuroprotective effect against inflammatory damage. Moreover, apigenin treatment after IL-1β damage was able to protect the entire neurite network and increased the intensity of TUBB3 labeling with neuron cluster formation [53]. In another study, we used in vitro studies based on NSCs isolated from the SVZ of postnatal balb/c mice to investigate the effect of troxerutin (TRX) on individual neurogenesis processes in general and specifically its neuroprotective effect against amyloid beta 1–42-induced inhibition of differentiation. An analysis was carried out. TRX increased neuronal differentiation of NSCs in a dose-dependent manner and significantly increased neurite outgrowth after 48 h and 7 days of incubation. A higher concentration of TRX also neutralized the inhibitory effects of amyloid beta 42 on neurite growth and length after 48 h of incubation. TRX significantly stimulated cell migration. Overall, TRX not only promoted the differentiation and migration of NSCs but also neutralized the inhibitory effects of amyloid beta 42 on NSCs [50]. Astragalus flavone therapy was capable of reducing the neurological function scores and cerebral infarct volume of the cerebral infarction model. Furthermore, astragalus therapy was able to increase BrdU-positive cells and reduce GFAP expression while promoting the expression of nestin, TUBB3, and O4. In hypoxia condition, TUBB3 expression after ASF treatment is lower than that in the hypoxia model. In a study closest to the study we conducted, the application of naringenin to the cell culture of the airway fluid of rats exposed to cigarette smoke increased beta tubulin IV levels. In our study, the fact that naringin supplementation after I/R for 2 weeks increased beta tubulin values is similar to the studies mentioned above [27].

Finally, in our study, the effect of brain I/R and 2-week naringin application on NeuN levels, which is an important factor in neurogenesis, in ovariectomized rats was examined. While the NeuN value showed a significant decrease with ischemia–reperfusion, 2-week naringin supplementation led to a significant increase in this parameter in both the hippocampus and frontal cortex. NeuN is a neuronal nuclear protein found in the nuclei and perinuclear cytoplasm of neurons [10]. NeuN is found in post-mitotic neurons and often persists in mature neurons [54]. In neurological research, the expression level of NeuN is used to evaluate neuron death [9]. In a study, it was confirmed that hesperidin flavonoid treatment prevented the decrease in neural cell survival along with the improvement in NeuN-positive cell loss in rats with memory impairment associated with hippocampal neurogenesis [10]. NeuN is widely used in stroke research because it is a stable and reliable marker of mature neurons [55]. In a study, the effect of focal cerebral I/R on the expression of NeuN protein was examined, and it was observed that the number of NeuN-positive cells decreased compared to the sham group [56]. In one study, a common ultramicronized compound consisting of N-palmitoylethanolamine, an endogenous fatty acid amide member of the N-acylethanolamine family, combined with the plant flavonoid luteolin, called co-ultra PEALut, was found to be effective in a mouse model of vascular dementia 15 days after MCAO. Its neuroprotective properties were evaluated. Immunofluorescence staining showed that carotid artery ligation reduced NeuN immune content 15 days after surgery, while co-ultra PEALut treatment repaired it by increasing the number of neuronal cells. Recent studies revealed that baicalin, a flavonoid compound derived from the root of *Scutellaria baicalensis* Georgi, could promote neuronal differentiation of NSPCs after initiating the differentiation process in vitro. BrdU/NeuN double-staining analysis showed that baicalin could promote new neuron production after cerebral ischemia [57]. *Alpinia katsumadai* seed extract (EAKS) has been reported to have anti-inflammatory effects and increase antioxidant activities. Neuroprotective effects of EACS against ischemic damage were observed in gerbils administered EACS orally once a day for 7 days before transient cerebral ischemia. In the EACS-treated ischemia group, there was a significant increase in NeuN-immunoreactive pyramidal neurons in the hippocampal CA1 region 4 days after I/R compared to the vehicle-treated ischemia group [44].

The effects of astragaloside VI on inducing NSC proliferation and self-renewal in vitro and enhancing neurogenesis for recovery of neurological functions in post-ischemic brains in vivo were investigated. For animal experiments, rats underwent MCAO for 1.5 h and reperfusion for 7 days. Astragaloside VI was administered via intravenous injection daily for 7 days. Astragaloside VI treatment promoted neurogenesis and estrogenic formation in the DG region, SVZ, and cortex of transient ischemic rat brains in vivo [58]. In another study, forebrain ischemia was performed by occlusion of four vessels. Baicalin has been shown to significantly increase the number of newly generated cells in the DG of the hippocampus after transient forebrain ischemia. Baicalin-treated animals exhibited an increase in the fraction of BrdU/NeuN double-positive cells compared to vehicle-treated animals. In conclusion, this study demonstrated that baicalin can prepare NSPCs towards a neuronal fate and facilitate subsequent differentiation into neurons, and stimulate adult hippocampal neurogenesis after cerebral ischemic injury [59].

Similar to our research, Yılmaz et al. when naringin supplementation was administered to male rats at the same dose for 2 weeks after I/R, it significantly increased Neu N values in the hippocampus and frontal cortex and significantly corrected the decrease in this parameter due to I/R. In our study, the application of naringin at the same dose for 2 weeks after ovariectomy in female rats increased the Neu N values, which decreased due to I/R, and even exceeded the control values, revealing that the positive effect of naringin supplementation does not differ depending on gender [60].

### Limitations

This study was conducted exclusively on female rats, and the potential effects on male rats were not investigated.

Neurological scoring was utilized; however, assessments of learning, memory, and cognitive functions were not performed.

Only a single dose and administration duration of naringin were evaluated in this study. Further research is required to assess the effects of varying doses and administration periods.

The absence of direct cerebral blood flow measurements and the lack of additional neurobehavioral tests are considered limitations of the present study. Future research may incorporate these methods to provide a more comprehensive validation of the ischemia–reperfusion model.

## Conclusion

Looking at the results of the current research, first of all, it is observed that neurological disability occurs due to I/R.

Again, as a result of I/R, calbindin values, which have a protective effect on neurons, and tubulin and NeuN levels, which are effective in neurogenesis, were significantly suppressed.

However, intraperitoneal naringin supplementation for 2 weeks had a positive effect on neurological failure and neurogenesis due to I/R in ovariectomized rats.

In future studies, the mechanism can be explained in more detail by investigating different experimental periods and application times, and the molecules in these pathways.

## Data Availability

The datasets which were generated during the current study are available from the corresponding author on reasonable request.
